# Real world outcomes and prognostic factors in Chinese patients with primary mediastinal B cell lymphoma: A single center experience and the Surveillance, Epidemiology, and End Results validation

**DOI:** 10.1007/s00277-026-06914-4

**Published:** 2026-02-28

**Authors:** Boya Wang, Ruize Chen, Huanhuan Wang, Tonglu Qiu, Zihan Liang, Lei Cao, Huayuan Zhu, Wei Xu, Lei Fan, Jianyong Li, Yi Miao, Yi Xia

**Affiliations:** Department of Hematology, Jiangsu Province Hospital, The First Affiliated Hospital with Nanjing Medical University, Nanjing, 210000 China

**Keywords:** Primary mediastinal B cell lymphoma, Cancer treatment, Prognosis, Chemotherapy, Radiotherapy

## Abstract

This study aimed to characterize the clinical characteristics, treatment patterns, survival outcomes, and prognostic factors of Chinese patients with primary mediastinal B-cell lymphoma (PMBCL). We retrospectively reviewed 69 consecutive patients with PMBCL treated at our center between 2010 and 2025. External validation was performed using data from the Surveillance, Epidemiology, and End Results (SEER) database (n = 1,460). The median age at diagnosis was 33 years (range, 18–62), with a female predominance (female-to-male ratio, 2.1:1). Most patients (92.8%, 64/69) received R-DA-EPOCH as frontline therapy, and 29.0% (20/69) additionally underwent consolidative radiotherapy (RT). Among 51 evaluable patients, the objective response rate was 90.2%, with a complete response rate of 74.5%. After a median follow-up of 74 months (range, 2–166), the estimated 5-year progression-free survival (PFS) and overall survival (OS) rates in whole cohort were 88.7% and 94.8%, respectively. Hematologic toxicity, predominantly febrile neutropenia, was the most common adverse event; nevertheless, 60.4% of patients maintained a relative dose intensity ≥ 100%. Consolidative RT did not confer a survival advantage, whereas age > 40 years emerged as an adverse prognostic factor for OS, a finding further validated in the SEER cohort. Chinese patients with PMBCL generally have favorable outcomes in the modern treatment era. However, older age (> 40 years) is associated with inferior survival. The R-DA-EPOCH regimen demonstrated high efficacy in this real-world setting, whereas additionally consolidative RT failed to provide a significant survival benefit.

**Supplementary Information:**

The online version contains supplementary material available at 10.1007/s00277-026-06914-4.

## Introduction

Primary mediastinal large B-cell lymphoma (PMBCL) is a distinct subtype of non-Hodgkin lymphoma originating from thymic B cells and accounts for approximately 2–4% of all cases [1]. It predominantly affects younger individuals, with a median age at diagnosis of around 35 years, and shows a marked female predominance (male-to-female ratio of approximately 1:3) [2, 3]. Clinically, PMBCL typically presents as a bulky mass in the anterior superior mediastinum (≥ 10 cm), frequently invading adjacent structures such as the lungs, pleura, and pericardium, and resulting in compressive symptoms including superior vena cava syndrome, pleural effusion, or pericardial effusion. At diagnosis, more than 80% of patients have early-stage disease (stage I–II), whereas distant nodal involvement or bone marrow infiltration is uncommon [4–6].

There is currently no universally accepted standard first-line therapy for PMBCL. Early treatment strategies were largely extrapolated from diffuse large B-cell lymphoma and centered on rituximab-containing, anthracycline-based chemotherapy, most commonly R-CHOP (rituximab, cyclophosphamide, doxorubicin, vincristine, and prednisone) [7]. However, multiple retrospective studies conducted between 1990 and 2010 demonstrated that the 2-year relapse rate for R-CHOP without consolidative radiotherapy (RT) reached 30%–40% [8–11]. More recently, the IELSG37 trial demonstrated that R-CHOP21 resulted in a higher proportion of patients with Deauville score (DS) 5 compared with alternative regimens (24% vs. 8%), accompanied by smaller reductions in metabolic tumor volume (MTV) and less pronounced declines in post-treatment SUVmax, suggesting suboptimal disease control with R-CHOP21 in PMBCL [12]. In contrast, a prospective phase II study (CALGB 50303) reported that DA-EPOCH-R (dose-adjusted etoposide, prednisone, vincristine, cyclophosphamide, doxorubicin, and rituximab) achieved a 5-year event-free survival (EFS) of 93% and an overall survival (OS) of 97%, while allowing omission of RT in more than 70% of patients. Nevertheless, this intensified regimen was associated with substantially higher rates of hematologic and infectious toxicities [13–15].

Previous studies suggested that consolidative RT may reduce the risk of local relapse; however, particularly in younger patient populations, RT is associated with an increased risk of long-term cardiac toxicity and secondary malignancies [16, 17]. End-of-treatment positron emission tomography/computed tomography (EOT-PET/CT) has therefore emerged as a critical tool for response assessment in PMBCL, guiding decisions regarding the use of consolidative RT and helping to minimize unnecessary radiation exposure [17–19]. The phase III randomized IELSG37 trial demonstrated that omission of RT did not compromise survival outcomes in patients who achieved complete metabolic remission (Deauville score [DS] 1–3) following first-line immunochemotherapy [20]. Consistent with these findings, the 2025 National Comprehensive Cancer Network (NCCN) Guidelines recommend reserving RT for patients with biopsy-proven residual disease or positive EOT-PET findings (DS 4–5), while observation is advised for all other patients [21].

Accordingly, we conducted a retrospective analysis of 69 patients with PMBCL treated at the First Affiliated Hospital with Nanjing Medical University (Jiangsu Province Hospital) between 2010 and 2025, together with 1,460 PMBCL patients identified from the Surveillance, Epidemiology, and End Results (SEER) database between 2000 and 2019. This study aimed to comprehensively evaluate the clinical characteristics, treatment patterns, survival outcomes, and prognostic factors of PMBCL, thereby providing real-world evidence to inform clinical practice.

## Materials and methods

### Study population

This study included 69 patients with PMBCL who received at least one cycle of chemoimmunotherapy at the First Affiliated Hospital with Nanjing Medical University (Jiangsu Province Hospital) between October 2010 and May 2025. All cases treated at our center were diagnosed by at least two experienced hematopathologists in accordance with the World Health Organization (WHO) Classification of Tumors of Haematopoietic and Lymphoid Tissues [22]. All patients had baseline clinical data and treatment records available. The study did not impose a minimum follow-up duration; and no minimum follow-up duration was required. For comparison, 1,460 PMBCL patients diagnosed between 2000 and 2019 were identified from the SEER database.

### Evaluation of response

Treatment response was assessed after completion of the third and sixth cycles of chemotherapy using PET/CT or contrast-enhanced CT scans of the neck, chest, and abdomen. Responses were evaluated according to the 2014 Lugano criteria [23], and classified as complete remission/complete metabolic remission (CR/CMR), partial remission/partial metabolic remission (PR/PMR), or progressive disease (PD). The objective response rate (ORR) was defined as the proportion of patients achieving CR/CMR or PR/PMR.

OS was defined as the time from diagnosis to death from any cause or last follow-up. Progression-free survival (PFS) was defined as the time from diagnosis to disease progression, relapse, death from any cause, or last follow-up.

### Statistical analysis

All statistical analyses were performed using IBM SPSS Statistics version 23.0. Categorical variables are presented as frequencies and percentages, while continuous variables are expressed as the median (range) or mean ± standard deviation, as appropriate. Comparisons between groups were conducted using the chi-square test or Fisher’s exact test. Survival curves were estimated using the Kaplan–Meier method. Univariate Cox proportional hazards regression was used to identify potential prognostic factors for PFS and OS. Variables with a *p* value < 0.20 in univariate analysis were entered into multivariate Cox proportional hazards models to determine independent prognostic factors, with results reported as hazard ratios (HRs) and 95% confidence intervals (CIs).

## Results

### Patient characteristics

A total of 69 patients with PMBCL were included, with a median age of 33 years (range, 18–62) and 47 (68.1%) females, resulting in a female-to-male ratio of 2.1:1. Ten patients (14.5%) had an Eastern Cooperative Oncology Group performance status (ECOG-PS) ≥ 2. According to the Ann Arbor staging system, 15.9% (11/69) were classified as stage III–IV. Extranodal involvement—including lung, kidney, gastrointestinal tract, bone, or bone marrow—was observed in 27.9% (19/68) of patients. A bulky mediastinal mass (≥ 10 cm) was present in 38.6% (22/57). Regarding prognostic indices, 27.5% (19/69) had an International Prognostic Index (IPI) ≥ 2, and 40.6% (28/69) had an NCCN-IPI ≥ 2.

### Induction treatment

The majority of patients (92.8%, 64/69) received R-DA-EPOCH as first-line therapy, while 7.2% (5/69) were treated with R-CHOP. Consolidative RT (36–45 Gy), administered after completion of chemotherapy, was given to 29.0% (20/69) of patients, with detailed RT characteristics provided in the Supplementary Table 1. Additionally, 22.1% (15/68) underwent surgical resection of the mediastinal mass prior to systemic chemotherapy (Table 1).

**Table 1:** Clinical characters for PMBCL patients in the in-house cohort

Characteristics		Total (*n* = 69)
Sex, n(%)		
	Female	47/69 (68.1)
	Male	22/69 (31.9)
Age at diagnosis, years		
Median (Range)		33 (18–62)
Age categories, n (%)		
	≤ 40 years	57/69 (82.6)
	> 40 years	12/69 (17.4)
Albumin, n (%)		
	Normal	34/67 (50.7)
	< 40 g/L	33/67 (49.3)
Serum LDH level, n (%)		
	Normal	25/69 (36.2)
	> 250 mmol/L	44/69 (63.8)
ECOG-PS, n (%)		
	0–1	59/69 (85.5)
	≥ 2	10/69 (14.5)
B symptoms, n (%)		14/69 (20.3)
Stage, n (%)		
	I/II	58/69 (84.1)
	III/IV	11/69 (15.9)
Extranodal involvement, n (%)		
	No	49/68 (72.1)
	Yes	19/68 (27.9)
Pleural effusion, n (%)		19/69 (27.5)
Pericardial effusion, n (%)		8/69 (11.6)
Tumor size at diagnosis, n (%)		
	< 10 cm	35/57 (61.4)
	≥ 10 cm	22/57 (38.6)
Thrombogenesis, n (%)		5/69 (7.2)
IPI, n (%)		
	0–1	50/69 (72.5)
	≥ 2	19/69 (27.5)
NCCN-IPI, n (%)		
	0–1	41/69 (59.4)
	≥ 2	28/69 (40.6)
RT, n (%)		
	No	49/69 (71.0)
	Yes	20/69 (29.0)
Surgery, n (%)		
	No	53/68 (77.9)
	Yes	15/68 (22.1)
Chemotherapy, n (%)		
	R-CHOP	5/69 (7.2)
	R-DA-EPOCH	64/69 (92.8)

(*ECOG* Eastern Cooperative Oncology Group Performance Status, *NCCN-IPI* National Comprehensive Cancer Network-IPI, *IPI* International Prognostic Index, RT radiotherapy, *R-DA-EPOCH* dose adjusted etoposide-prednisone-vincristine-cyclophosphamide-doxorubicin and rituximab)

### Therapeutic effect evaluation and survival

Two populations were defined for efficacy assessment. The entire cohort of 69 patients comprised the intention-to-treat (ITT) population, while 51 patients (73.9%) constituted the evaluable population for efficacy (EPP). Patients were excluded from the EPP due to unavailable end-of-treatment assessments (*n* = 11), incomplete treatment at our institution (*n* = 6), or death prior to response evaluation (*n* = 1). Response evaluation was performed using PET/CT in 49 patients (96.1%) and contrast-enhanced CT in 2 patients (3.9%), with detailed imaging criteria provided in Supplementary Table 2.

Within the EPP (*n* = 51), the ORR was 90.2% (46/51), including a CR/CMR rate of 74.5% (38/51). Patients treated with R-DA-EPOCH demonstrated higher ORR and CR/CMR rates in the EPP, as well as superior 5-year PFS in the ITT population compared with those receiving R-CHOP, despite the small size of the R-CHOP group. The addition of consolidative RT did not improve 5-year PFS or OS in the overall R-DA-EPOCH cohort (Table 2).

**Table 2:** Efficacy assessment of different treatment regimens

Characteristics^$^		ORR, %	CR/CMR, *n*(%)	PR/PMR, *n*(%)	5-year PFS, %	5-year OS, %
Total (*n* = 51)		90.2	38 (74.5)	8 (15.7)	89.2	97.2
R-CHOP (*n* = 3)		33.3	1 (33.3)	0 (0.0)	66.7	100
R-DA-EPOCH (*n* = 48)		93.8	37 (77.1)	8 (16.7)	90.7	97.1
	Non-RT (*n* = 33)	97.0	27 (81.8)	5 (15.2)	92.4	95.5
	RT (*n* = 15)	86.7	10 (66.7)	3 (20.0)	86.7	100
	*p* value	0.227^#^			0.750*	0.460*
**Characteristics** ^&^		**5-year PFS**,**%**			**5-year OS**,** %**	
Total (*n* = 69)		88.7			94.8	
R-CHOP (*n* = 5)		80.0			100	
R-DA-EPOCH (*n* = 64)		89.5			94.4	
	Non-RT (*n* = 46)	89.8			92.0	
	RT (*n* = 18)	88.9			100	
	*p* value	0.956*			0.258*	

(^$^These numbers represent the subgroup of the EPP, ^¥^These numbers represent the subgroup who received only *R-DA-EPOCH* chemotherapy in the EPP, ^&^These numbers represent the ITT population, ^%^These numbers represent the subgroup who received only *R-DA-EPOCH* chemotherapy in the ITT population, ^#^Fisher exact test, *Log-rank test)

All patients were followed until May 1, 2025, with a median follow-up of 74 months (range, 2–166). In the entire cohort, the estimated 5-year PFS and OS rates were 88.7% and 94.8%, respectively, with neither median PFS nor median OS reached.

Univariate analysis in the entire cohort (*n* = 69) identified age > 40 years (*p* = 0.036) and bulky tumor (diameter ≥ 10 cm; *p* = 0.047) as adverse prognostic factors for PFS, whereas treatment with R-DA-EPOCH was associated with improved PFS (*p* = 0.020). Multivariate Cox regression confirmed that only R-DA-EPOCH treatment remained an independent predictor of PFS. For OS, univariate analysis showed age > 40 years (*p* = 0.009) as an adverse factor and R-DA-EPOCH as favorable (*p* = 0.042), with multivariate analysis showing both as independent prognostic factors. Notably, consolidative RT was not identified as an independent predictor of either PFS or OS in univariate or multivariate analyses, suggesting limited survival benefit in this cohort (Table 3).

**Table 3:** Univariate and multivariate analyses of progression-free and overall survival of the entire cohort

Characteristics	Total (*N*)	Univariate-PFS		Mutivariate-PFS		Univariate-OS		Mutivariate-OS	
		Hazard ratio (95% CI)	*p* value	Hazard ratio (95% CI)	*p* value	Hazard ratio (95% CI)	*p*-value	Hazard ratio (95% CI)	*p*-value
Sex	69								
Female	47	Reference		Reference		Reference			
Male	22	0.39 (0.11 ~ 1.37)	0.142	0.70 (0.12 ~ 3.98)	0.689	1.67 (0.19 ~ 15.00)	0.645		
Age	69								
≤ 40 years	57	Reference		Reference		Reference		Reference	
> 40 years	12	3.93 (1.10 ~ 14.11)	0.036	4.14 (0.82 ~ 20.83)	0.085	11.36 (1.85 ~ 69.70)	0.009	30.13 (2.61 ~ 347.27)	0.006
Albumin	67								
Normal	34	Reference				Reference			
< 40 g/L	33	2.22 (0.55 ~ 8.91)	0.260			3.16 (0.33 ~ 30.42)	0.320		
Serum LDH level	69								
Normal	25	Reference		Reference		Reference			
> 250 mmol/L	44	5.64 (0.71 ~ 44.63)	0.101	8.65 (0.60 ~ 124.61)	0.113	2.33 (0.26 ~ 20.92)	0.452		
ECOG	69								
0–1	59	Reference				Reference			
≥ 2	10	1.98 (0.41 ~ 9.61)	0.395			2.42 (0.25 ~ 23.16)	0.443		
B symptoms	69								
No	55	Reference				Reference			
Yes	14	1.74 (0.45 ~ 6.76)	0.422			1.04 (0.12 ~ 9.41)	0.969		
Stage	69								
I/II	58	Reference				Reference			
III/IV	11	1.18 (0.25 ~ 5.56)	0.835			1.14 (0.13 ~ 10.22)	0.906		
Extranodal involvement	68								
No	49	Reference				Reference			
Yes	19	0.66 (0.14 ~ 3.12)	0.602			0.68 (0.08 ~ 6.10)	0.731		
Pleural effusion	69								
No	50	Reference							
Yes	19	1.58 (0.44 ~ 5.62)	0.479			1.62 (0.27 ~ 9.72)	0.597		
Pericardial effusion	69								
No	61	Reference							
Yes	8	0.81 (0.10 ~ 6.39)	0.839			1.92 (0.21 ~ 17.36)	0.562		
Tumor size at diagnosis	57								
< 10 cm	35	Reference		Reference		Reference			
≥ 10 cm	22	5.07 (1.02 ~ 25.18)	0.047	2.02 (0.30 ~ 13.81)	0.474	3.03 (0.27 ~ 33.72)	0.367		
IPI	69								
0–1	50	Reference				Reference			
≥ 2	19	2.04 (0.57 ~ 7.28)	0.272			2.16 (0.36 ~ 13.17)	0.402		
RT	69								
No	49	Reference				Reference			
Yes	20	1.49 (0.42 ~ 5.28)	0.539			0.47 (0.05 ~ 4.25)	0.503		
Surgery	68								
No	53	Reference				Reference			
Yes	15	0.37 (0.05 ~ 2.92)	0.345			0.86 (0.10 ~ 7.73)	0.891		
Chemotherapy	69								
R-CHOP	5	Reference		Reference		Reference		Reference	
R-DA-EPOCH	64	0.20 (0.05 ~ 0.78)	0.020	0.09 (0.01 ~ 0.83)	0.034	0.15 (0.03 ~ 0.94)	0.042	0.05 (0.00 ~ 0.55)	0.014

(*ECOG* Eastern Cooperative Oncology Group Performance Status, *HR* hazard ratio, *IPI* International Prognostic Index, *OS* overall survival, *PFS* progression-free survival)

### Safety assessment

Given the retrospective nature of this study, all safety and dose-intensity data are provided for reference only. Adverse events (AEs) were graded according to the Common Terminology Criteria for Adverse Events (CTCAE) version 5.0. R-DA-EPOCH was associated with a high incidence of hematologic toxicity (60.9%), predominantly neutropenia, while pneumonia was the most frequent non-hematologic AE. No treatment-related deaths were observed (Supplementary Table 3).

Treatment dose information was unavailable for four patients, and two patients did not complete all six planned cycles due to death or early autologous hematopoietic stem cell transplantation (ASCT). Relative dose intensity (RDI) was calculated for 63 evaluable patients (91.3%). Among R-DA-EPOCH patients (*n* = 58), the median RDI was 100% (range, 75.0%–127.9%), with dose reductions required in 39.6%, most commonly due to febrile neutropenia; nevertheless, 60.4% of patients were able to complete treatment with a RDI of ≥ 100%. In the R-CHOP group, the median RDI was also 100% (range, 90.0%–100%), with dose reductions in 40.0% of patients (Supplementary Table 4).

### Treatment outcomes in non-complete responders

Among the eight patients (15.7%) who achieved a PR after initial therapy, three received autologous stem cell transplantation (ASCT) following consolidative RT, and three underwent ASCT directly; all six subsequently achieved CR on PET/CT. Follow-up information was unavailable for the remaining two patients.

Of the five patients (9.8%) with progressive disease, one died from persistent progression after DHAP therapy (dexamethasone, cytarabine, cisplatin). One patient received chimeric antigen receptor T-cell (CAR-T) therapy following RT, another underwent ASCT after BV-R-GOD (Brentuximab vedotin, Rituximab, Gemcitabine, Oxaliplatin, Dexamethasone) combined with RT, and a third received a PD-1 inhibitor plus R-GemOx (Rituximab, Gemcitabine, Oxaliplatin) and RT; all three subsequently achieved and remain in CR. Treatment details for one additional patient were unavailable. Notably, two patients (2.9%) developed myelodysplastic syndrome (MDS) following ASCT.

### SEER database validation analysis

**Fig. 1:**
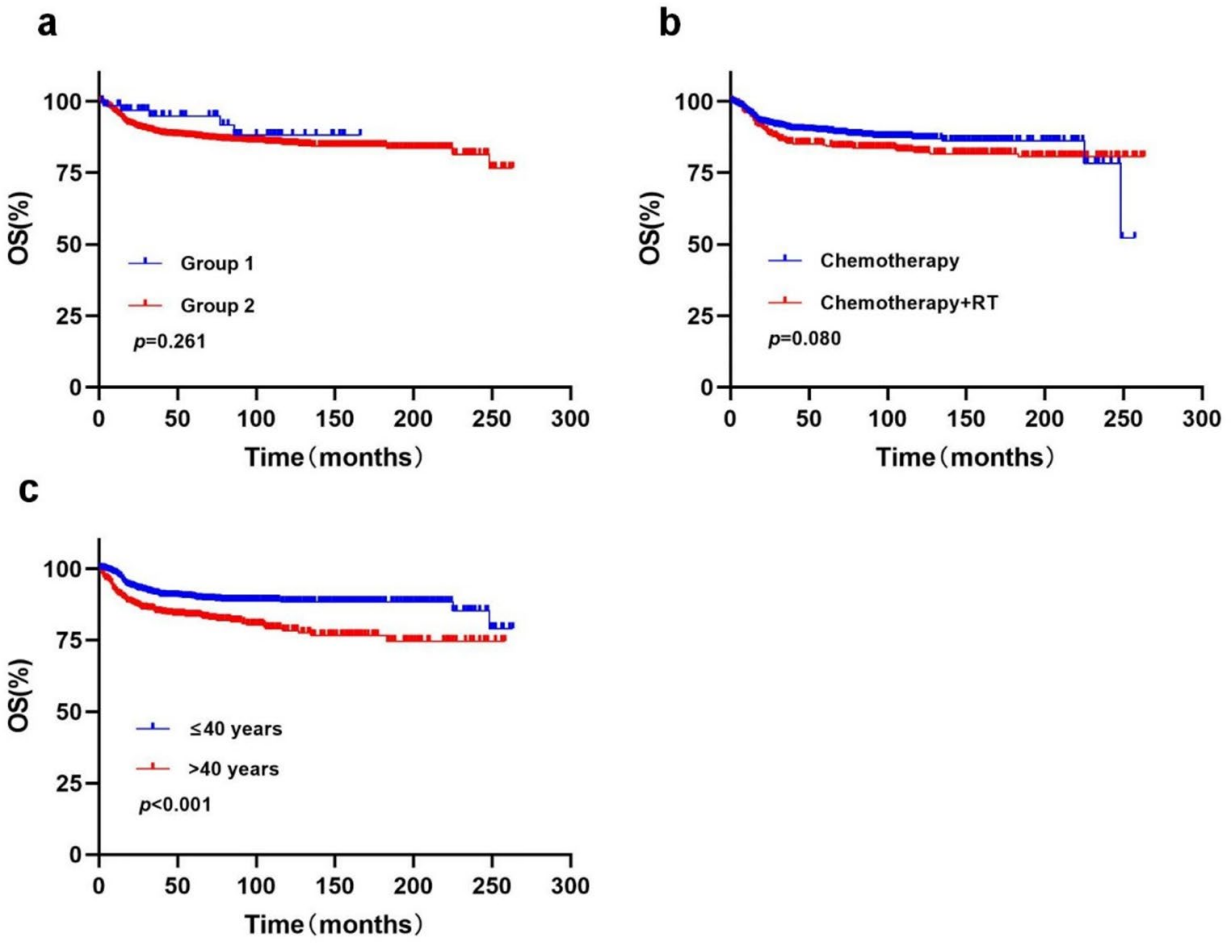
Comparison of survival outcomes using the SEER database for external validation (**a**) Overall survival (OS) comparing the single-center cohort (Group 1, *n* = 69) with the SEER cohort (Group 2, *n* = 1,460) (**b**) OS comparing PMBCL patients in the SEER cohort who received radiotherapy (RT) in addition to chemotherapy (*n* = 370) with those treated with chemotherapy alone (*n* = 983). (Chemotherapy regimens in the SEER database are not specified.) (**c**) OS in the SEER cohort stratified by age at diagnosis: ≤40 years (*n* = 883) versus > 40 years (*n* = 577)

Given the limited sample size of our single-center cohort, validation was performed using data from the SEER database, which included 1,460 patients diagnosed with PMBCL. Comparison of OS curves showed no significant difference between our cohort and the SEER cohort (*p* = 0.261) (Fig. 1a). However, due to differences across historical periods and the lack of detailed information on treatment regimens and prognostic factors in the SEER database, the statistical power for direct comparisons between the two cohorts was limited.

Within the SEER cohort, 94.3% (1,377/1,460) of patients received chemotherapy, of whom 27.3% (370/1,353) also underwent RT. Survival analysis indicated a 5-year OS of 84.7% in the RT group versus 89.4% in the non-RT group, without a statistically significant difference (*p* = 0.080) (Fig. 1b). Age-stratified analysis confirmed that age > 40 years was associated with inferior survival, with 5-year OS rates of 89.8% for patients aged ≤ 40 years and 83.5% for those > 40 years (*p* < 0.001) (Fig. 1c). These findings are consistent with our single-center cohort, further establishing age as an important independent prognostic factor in PMBCL.

## Discussion

This retrospective analysis represents the largest single-center cohort of PMBCL in China to date, providing a comprehensive evaluation of the long-term efficacy of R-DA-EPOCH in a real-world setting and identifying clinical factors influencing survival outcomes. Frontline R-DA-EPOCH demonstrated high efficacy with manageable hematologic toxicity, while consolidative RT did not improve survival, and age > 40 years emerged as an independent adverse prognostic factor.

As expected, our cohort predominantly consisted of young female patients (median age 33 years) presenting with bulky mediastinal masses, most commonly detected due to dyspnea or chest discomfort, consistent with the characteristic clinical phenotype of PMBCL. Stage I/II disease accounted for 85.5% of cases, in line with previous reports [1, 16]. Despite its aggressive presentation, PMBCL is generally associated with a favorable prognosis [16, 24, 25], and in our study, 5-year PFS and OS exceeded 85% following first-line therapy.

Presently, no consensus exists regarding the optimal first-line therapy for PMBCL. In our study, patients receiving R-DA-EPOCH achieved higher ORR and CR/CMR rates in the EPP, as well as superior 5-year PFS in the overall cohort compared with those receiving R-CHOP, consistent with the CALGB 50303 trial (5-year EFS 93%, OS 97%) [14]. Similarly, the IELSG-37 analysis reported a significantly higher rate of DS 5 after R-CHOP21 compared with other regimens (23.8% vs. 8.2%), suggesting suboptimal metabolic remission [12]. Although the number of R-CHOP-treated patients in our cohort was limited, accumulating real-world evidence indicates that R-DA-EPOCH can achieve deeper metabolic remission and may reduce the need for consolidative RT in patients able to tolerate intensified therapy [4, 15].

This potential benefit must, however, be weighed against the increased risk of acute toxicity associated with dose-intensified regimens, particularly hematologic adverse events such as febrile neutropenia [14]. Consistent with previous reports, our cohort experienced a high incidence of hematologic toxicity in the R-DA-EPOCH group, predominantly febrile neutropenia [26, 27]. These findings underscore the importance of close monitoring of hematologic parameters and infectious complications, as well as proactive supportive care. Routine prophylactic measures—including long-acting granulocyte colony-stimulating factor (G-CSF) for neutropenia, trimethoprim-sulfamethoxazole for antimicrobial prophylaxis, and acyclovir for viral prevention—may reduce treatment interruptions, lower infection-related hospitalizations, and help maintain therapeutic dose intensity [21, 28, 29].

Importantly, our study demonstrated comparable efficacy between R-DA-EPOCH with and without consolidative RT, with no statistically significant differences in survival outcomes. This observation was further supported by the SEER analysis, which showed no OS benefit with the addition of RT compared to chemotherapy alone (84.7% vs. 89.4%, *p* = 0.080), suggesting limited long-term impact of RT. Accordingly, the decision to administer consolidative RT should be individualized, incorporating end-of-treatment metabolic response (e.g., Deauville score) and patient-specific risk factors. PET/CT at the end of treatment is a critical tool for assessing disease distribution and therapeutic response, facilitating safe RT omission in patients achieving CR/CMR [30, 31]. The phase III IELSG-37 randomised trial confirmed that patients achieving complete metabolic remission (DS 1–3) after first-line immunochemotherapy maintained an excellent 3-year PFS of 96.2% without RT [20]. Among 48 patients treated with R-DA-EPOCH who underwent end-of-treatment evaluation, 27 achieved CR/CMR without consolidative RT and remained relapse-free at 5 years. This further supports the feasibility and effectiveness of PET-guided treatment decision-making in PMBCL.

However, both our cohort and the SEER database analysis demonstrated significantly inferior 5-year OS in patients older than 40 years (89.8% vs. 83.5%, *p* < 0.001), consistent with age-stratified analyses reported by Zhou et al. using SEER 18 [32]. Older age may represent an independent adverse prognostic factor, potentially due to reduced stem cell reserves and decreased tolerance to anthracycline-based regimens, limiting the ability to deliver full-dose intensive therapy [14, 33]. For elderly patients, modified treatment strategies that balance safety and efficacy are warranted. For example, the R-COMP-DI regimen, incorporating novel cytotoxic agents such as non-polyethylene glycol–modified liposomal doxorubicin, has shown promising preliminary results, achieving a CMR rate of 93% without requiring hospitalization [34].

Among the five patients with primary progression, three achieved sustained CR following second-line therapies, including CD30- and PD-1-targeted agents, ASCT, or CAR-T therapy, highlighting the importance of effective salvage strategies for refractory/relapsed disease. Notably, two patients (2/69, 2.9%) developed secondary MDS following ASCT, emphasizing that while ASCT can enhance remission and long-term survival, it carries a potential leukemogenic risk. Previous studies have reported therapy-related MDS incidence of 1–5% among lymphoma patients receiving high-dose chemotherapy and stem cell transplantation, primarily associated with cumulative DNA damage from cytotoxic agents (e.g., cyclophosphamide, cytarabine) or RT [35–38]. These findings underscore the need for long-term hematologic surveillance to enable early detection and timely intervention.

Several limitations of this study should be acknowledged. The single-center design and relatively small sample size, particularly in the R-CHOP subgroup (*n* = 5), limit the robustness of comparative analyses between treatment regimens. The retrospective nature of the study may also introduce selection bias, especially in treatment choice and RT decisions, which were determined at the discretion of treating physicians. On the other hand, the SEER database comparison has additional inherent limitations. The cohorts span different historical periods and may vary in diagnostic practices, treatment standards, and supportive care, providing only a limited external benchmark rather than direct equivalence. Despite these constraints, this study offers valuable real-world evidence on the long-term efficacy of R-DA-EPOCH in Chinese PMBCL patients and supports the feasibility of PET-guided RT omission, reinforced by external validation from the SEER database. Collectively, these findings provide clinically meaningful insights that may inform contemporary management strategies for PMBCL.

## Conclussion

In this real-world Chinese cohort, PMBCL predominantly affected young female patients and was associated with a favorable overall prognosis. Advanced age (> 40 years) emerged as an independent adverse prognostic factor. The R-DA-EPOCH regimen demonstrated high efficacy and sustained long-term survival. The addition of consolidative RT did not confer a significant survival benefit. Decisions regarding RT should be individualized based on clinical risk factors and PET/CT–assessed metabolic response. For high-risk patients, tailored salvage strategies and precise metabolic evaluation remain critical for optimizing treatment outcomes.

## Supplementary Information

Below is the link to the electronic supplementary material.

Supplementary Material 1

## Data Availability

All presented data and codes in this study are available from the corresponding author upon reasonable request.

## Declarations

### Permission to Reproduce Material From Other Sources

No previously published material was reproduced in this manuscript.

### Clinical Trial Registration (including trial number)

Patients were not included in any clinical trial.
