# Supplementary material for: Real world outcomes and prognostic factors in Chinese patients with primary mediastinal B cell lymphoma: A single center experience and the Surveillance, Epidemiology, and End Results validation
Source: Ann Hematol. 2026 Feb 28;105(4):147. doi: 10.1007/s00277-026-06914-4 (PMC12950073; doi:10.1007/s00277-026-06914-4)
Supplement: Supplementary file 1 — Supplementary Material 1 [file 277_2026_6914_MOESM1_ESM.docx]

| **Patient ID** | **Size of Residual Lymph Nodes** | **Site of Residual Lymph Nodes** | **Radiotherapy Dose** | **Radiotherapy Site** |
| --- | --- | --- | --- | --- |
| P6 |  |  | 40GY/20F |  |
| P11 | 1.1cm*1.4cm | mediastinum |  |  |
| P15 | 1.6cm | clavicle, neck | 36GY/20F | The lymph drainage area of the neck and mediastinum |
| P17 | 1.3cm*0.6cm | mediastinum | 36GY/20F | mediastinum |
| P18 |  |  | 40GY/20F |  |
| P20 | 5.2cm*2.4cm |  | 36GY/18F | mediastinum |
| P27 | 1.7cm*2.2cm | mediastinum | 40GY/20F | mediastinum |
| P29 | 3.3cm*1.5cm | clavicle, neck | 36GY/18F | The lymph drainage area of the neck and mediastinum |
| P32 | 0.6cm*0.71cm | clavicle, neck | 40GY/20F | The lymph drainage area of the neck and mediastinum |
| P33 | 2.9cm*1.9cm | mediastinum |  | mediastinum |
| P41 | 2.3cm*1.8cm | mediastinum | 50GY/25F | mediastinum |
| P42 | 1.5cm*1.5cm | neck, mediastinum, armpit | 45GY/25F | The lymph drainage area of the neck and mediastinum |
| P46 | 3.8cm*3.2cm | Mediastinum, clavicle | 36GY/18F | The lymph drainage area of the neck and mediastinum |
| P48 | 0.9cm*1.7cm | clavicle, neck, mediastinum | 45GY/25F | mediastinum |
| P50 | 1.2cm*1.8cm |  | 45GY/25F | mediastinum |
| P52 | 1.6cm*1.3cm | mediastinum |  |  |
| P55 | 1.1cm*0.8cm | mediastinum | 38GY/20F | mediastinum |
| P58 | 2.4cm | mediastinum, armpit | 36GY/11F | mediastinum |
| P63 | 1.9cm*0.7cm | mediastinum | 36GY/20F | mediastinum |
| P69 |  | mediastinum |  | mediastinum |

**Supplementary Table 1 RT Characteristics of 20 Patients**

**Supplementary Table 2 End-of-Treatment Imaging Evaluation Methodology**

| ****[¹⁸F]FDG PET/CT**** | | | **CT** |
| --- | --- | --- | --- |
| ****Scanner Models**** | Gemini GXL， UM780PET/CT， GE Discovery PET/CT Clarity 710 | | Siemens Healthineers NAEOTOM Alpha and Siemens Healthineers SOMATOM Force XCS |
| ****Response Criteria**** | **Lugano 2014 Classification** | | |
| ****Complete Metabolic Response (CMR)**** | Complete resolution of [¹⁸F]FDG uptake in all lesions on PET/CT, to a level ≤ mediastinal blood pool background or indistinguishable from surrounding normal tissue. FDG uptake in any residual mass must not be above background. | | The longest diameter of all measurable nodal lesions present before treatment is ≤1.5 cm, with no evidence of extranodal involvement. Previous extranodal lesions (e.g., liver, spleen, bone marrow) have completely resolved, and organ enlargement has returned to normal size. |
| ****Partial Metabolic Response (PMR)**** | Significant reduction in Tumor Metabolic Volume (TMTV) and Total Lesion Glycolysis (TLG) from baseline, with residual abnormal FDG uptake above mediastinal blood pool background. | | For up to six measurable nodal or extranodal lesions, the sum of the products of their longest diameters (SPD) has decreased by ≥50%. No new lesions have appeared. |
| ****Progressive Metabolic Disease (PMD)**** | Appearance of new FDG-avid lesions and/or a significant increase in the intensity and/or extent of FDG uptake in existing lesions. | | At least one of the following criteria must be met: ① For any single nodal or extranodal lesion, the longest diameter (LDi) is >1.5 cm and has increased by ≥50% from its nadir; ② For nodal or extranodal lesions with a longest diameter ≤2 cm, an increase of 0.5 cm in either the longest or shortest diameter; for lesions with a longest diameter >2 cm, an increase of 1 cm in either the longest or shortest diameter; ③ Splenomegaly, with the longest diameter of the spleen increasing by >50% from the baseline value (if there was no splenomegaly at baseline, an increase in splenic longest diameter >2 cm); ④ Appearance of new nodal lesions (any diameter >1.5 cm) or extranodal lesions (any diameter >1 cm), or clear regrowth of previously responsive lesions. |
| ****Deauville Score (DS)**** | **5-Point Scale** used for semi-quantitative assessment of target lesions | |  |
|  | **Score 1** | Uptake ≤ background |  |
|  | **Score 2** | Uptake ≤ mediastinal blood pool |  |
|  | **Score 3** | Uptake > mediastinal blood pool but ≤ liver |  |
|  | **Score 4** | Uptake moderately higher than liver |  |
|  | **Score 5** | Uptake markedly higher than liver and/or new lesions |  |
|  | **Score X** | New FDG uptake unlikely related to lymphoma |  |
| ****PET Scan Interpretation**** | **DS 1-3** | **Negative (Corresponding to CMR)** |  |
|  | **DS 4-5** | **Positive (Suggesting Active Disease)** |  |

| **Characteristics** | | | **R-DA-EPOCH (n=64)** | **R-CHOP-21 (n=5)** |
| --- | --- | --- | --- | --- |
| **Tatal, n (%)** | | | 39 (60.9) | 3 (60.0) |
| **Hematologic, n (%)** | | | 35 (54.7) | 3 (60.0) |
|  | Anemia, n (%) | | 6 (9.4) | 1 (20.0) |
|  | Neutropenia, n (%) | | 33 (51.6) | 3 (60.0) |
|  |  | Febrile Neutropenia, n (%) | 20 (31.3) | 2 (40.0) |
|  | Thrombocytopenia, n (%) | | 7 (10.9) | 2 (40.0) |
| **Non-hematologic, n (%)** | | | 5 (7.8) | 0 (0.0) |
|  | Hypokalemia, n (%) | | 1 (1.6) | 0 (0.0) |
|  | Elevated ALT/AST, n (%) | | 1 (1.6) | 0 (0.0) |
|  | Pneumonia, n (%) | | 3 (4.7) | 0 (0.0) |

**Supplementary Table 3** **Grade 3-4 hematologic and non-hematologic adverse events by treatment regimen (R-DA-EPOCH vs R-CHOP).**

(*ALT* alanine aminotransferase; *AST* aspartate aminotransferase)

**Supplementary Table 4 Treatment Dose Intensity and Density**

| **Characteristics** | | **R-DA-EPOCH**  **(n=64)** | **R-CHOP**  **(n=5)** |
| --- | --- | --- | --- |
| Completion of planned cycles, n (%) | | 58 (90.6) | 5 (100.0) |
|  | Median relative dose intensity (MRDI) (Range) | 100% (75.0%-127.9%) | 100% (90.0%-100%) |
|  | Patients requiring dose reduction, n (%) | 23 (39.7) | 2 (40.0) |
|  | **Primary reasons for dose reduction** | Febrile Neutropenia | Febrile Neutropenia |
| **Patients experiencing treatment discontinuation**, n (%) | | 6 (9.4) | 0 (0.0) |
